# A Short Peptide Derived from the ZorO Toxin Functions as an Effective Antimicrobial

**DOI:** 10.3390/toxins11070392

**Published:** 2019-07-04

**Authors:** Yuichi Otsuka, Tomohiro Ishikawa, Chisato Takahashi, Michiaki Masuda

**Affiliations:** 1 Department of Biochemistry and Molecular Biology, Graduate School of Science and Engineering, Saitama University, 255 Shimo-Okubo, Sakura-Ku, Saitama City, Saitama 388-8570, Japan; 2 Department of Microbiology, School of Medicine, Dokkyo Medical University, 880 Kitakobayashi, Mibu-machi, Shimotsuga-gun, Tochigi 321-0293, Japan

**Keywords:** Toxin–Antitoxin system, ZorO, antimicrobial peptide

## Abstract

Antimicrobial peptides are potential molecules for the development of novel antibiotic agents. The ZorO toxin of a type I toxin–antitoxin system in *Escherichia coli* O157:H7 is composed of 29 amino acids and its endogenous expression inhibits *E. coli* growth. However, little is known about its inhibitory mechanism. In this study, we demonstrate that the ZorO localized in the inner membrane affects the plasma membrane integrity and potential when expressed in *E. coli* cells, which triggers the production of cytotoxic hydroxyl radicals. We further show that five internal amino acids (Ala–Leu–Leu–Arg–Leu; ALLRL) of ZorO are necessary for its toxicity. This result prompted us to address the potential of the synthetic ALLRL peptide as an antimicrobial. Exogenously-added ALLRL peptide to Gram-positive bacteria, *Staphylococcus aureus* and *Bacillus subtilis*, and a fungus, *Candida albicans*, trigger cell membrane damage and exhibit growth defect, while having no effect on Gram-negative bacterium, *E. coli*. The ALLRL peptide retains its activity under the physiological salt concentrations, which is in contrast to natural antimicrobial peptides. Importantly, this peptide has no toxicity against mammalian cells. Taken together, an effective and short peptide, ALLRL, would be an attractive antimicrobial to Gram-positive bacteria and *C. albicans*.

## 1. Introduction

Antibiotic resistance remains a serious threat to human health and there is an urgent need for novel antimicrobial compounds with unique functional mechanisms. Many organisms including plants, insects, and humans produce peptides to escape from microbial infection [1]. Various microbes also produce antimicrobial peptides for their own survival [2]. In many cases, antimicrobial peptides target microbial cell membranes [1,3]. Because the compositions of cell membranes substantially differ between prokaryotes and eukaryotes, antimicrobial peptides display highly selective toxicity. Also, these peptides are less likely to promote resistance, as alterations of the lipid composition of cell membranes are often toxic to bacteria. These advantages have led to the development of therapeutic antimicrobial peptides [4], however, there are disadvantages that need to be overcome. For example, the antimicrobial activity of natural peptides is relatively low but their cytotoxicity against mammalian cells is high. Also, natural antimicrobial peptides are generally large, which are hard and expensive to synthesize or purify [5]. In addition, many peptides unfold and lose their antimicrobial activity in solution containing physiological salt concentrations [6,7,8]. Therefore, it is desirable to discover potent, yet smaller, less cytotoxic, and salt-insensitive antimicrobial peptides.

Toxin–Antitoxin (TA) systems are widely conserved in prokaryotic plasmids and chromosomes [9]. The functions of TA systems are being extensively investigated as more evidence links TA systems to plasmid maintenance [10,11], general stress response [12,13], and phage defense [14,15]. TA systems are composed of a stable toxin and a labile antitoxin that inhibits a harmful effect of the cognate toxin. They are mainly divided into two types, I and II, although there are actually six different types but only a few examples have been discovered in each type III, IV, V, and VI [16,17,18,19]. Toxins of type I TA systems are small hydrophobic proteins and their antitoxins are small non-coding RNAs that inhibit the translation of the cognate toxin mRNAs [20]. Fozo’s group previously identified and characterized a type I TA system, zorO-orzO, that is encoded in the genome of *Escherichia coli* O157:H7 [21]. The orzO antitoxin is a 174 nt non-coding RNA that inhibits *zorO* translation via base-pairing between orzO and 5′UTR of *zorO* mRNA [22]. The ZorO toxin is a small protein composed of 29 amino acids and its expression leads to growth arrest and/or cell death [21]. While ZorO has been proposed to be located at the cell membrane through its putative transmembrane domain, it remains unclear how ZorO exerts its toxicity.

To address the molecular mechanism of ZorO toxicity in *E. coli* cells, we investigated the distribution of ZorO, its effect on cell membranes, and the production of cytotoxic highly reactive oxygen species. In addition, we explored the antimicrobial activity of ZorO derivatives and identified a peptide composed of five amino acids with potent antimicrobial activity against Gram-positive bacteria and a fungus, *Candida albicans*.

## 2. Results

### 2.1. ZorO Affects the Plasma Membrane Potential and Integrity

**Figure 1:**
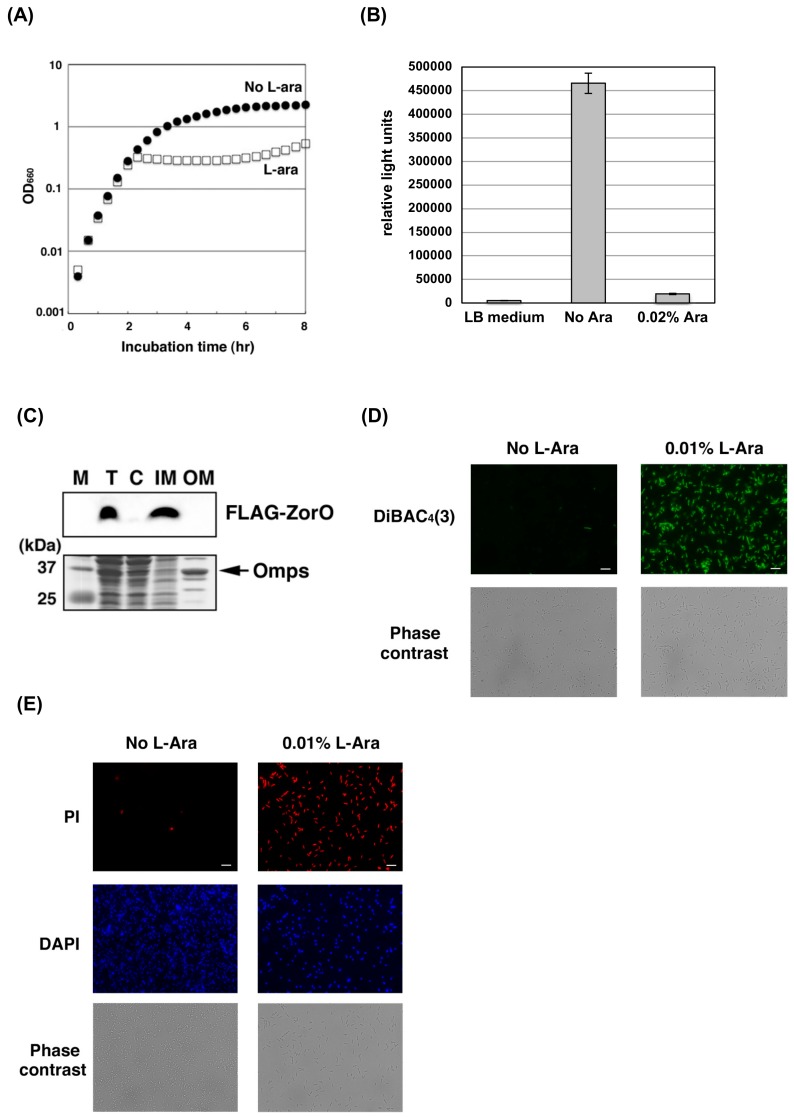
Toxicity of ZorO (**A**) *E. coli* TY0807 cells harboring pBAD24-FLAG-zorO were treated with (open squares) or without (closed circles) 0.1% arabinose (L-Ara). L-Ara was added to induce ZorO expression when the OD_660_ reached approximately 0.3. (**B**) TY0807 cells harboring pBAD24-FLAG-zorO were treated with 0.02% L-Ara for 30 min and diluted 10-fold with LB medium. Cell viability was determined using BacTiter-Glo™ Microbial Cell Viability Assay kit. The vertical axis indicates relative light units and the furthest left column is the value of LB medium only. (**C**) TY0807 cells harboring pBAD24-FLAG-zorO were treated with 0.1% L-Ara for 10 min. Cell lysate was fractionated as described in *Materials and Methods*. The upper or lower panel show the Western blot with anti-FLAG antibody or the CBB staining. M; marker, T; total cell lysate, C; cytoplasmic fraction, IM; inner membrane fraction, OM; outer membrane fraction. Omps indicates outer membrane protein C and F. (**D**) TY0807 cells harboring pBAD24-FLAG-zorO were incubated with (right panels) or without (left panels) 0.01% L-Ara for 30 min and then treated with a membrane potential-sensitive dye, DiBAC_4_(3). Upper or lower panels show DiBAC_4_(3) staining or phase contrast images. Scale bar is 10 µm. (**E**) TY0807 cells harboring pBAD24-FLAG-zorO were incubated with (right panels) or without (left panels) 0.01% L-Ara for 30 min and then treated with PI and DAPI. Upper, middle or lower panels show PI staining, DAPI staining, or phase-contrast images. Scale bar is 10 µm.

Consisting of 29 amino-acid residues, ZorO is one of the smallest toxins in the bacterial TA systems. To elucidate the mechanism of the ZorO toxicity, ZorO was expressed in *E. coli* K12 TY0807 cells. As reported previously [21], ZorO inhibited the growth of the *E. coli* cells (Figure 1A). It was also shown that the growth inhibition was associated with a significant reduction of the amount of ATP (Figure 1B), suggesting that the toxicity involves negative effects on the cellular metabolism.

To examine the subcellular distribution of ZorO, FLAG-tagged ZorO was expressed in *E. coli* TY0807 cells, and the cell lysates were fractionated and examined by Western blotting with an anti-FLAG antibody (Figure 1C). The result indicated that ZorO was located solely in the inner membrane fraction, compatible with the presence of a putative transmembrane helix [23,24]. We next examined whether the presence of ZorO affects the membrane potential by using a membrane potential-sensitive dye, DiBAC_4_(3), which enters the cells upon plasma membrane depolarization and increases the fluorescence intensity though binding to intracellular proteins or membranes [25]. When *E. coli* TY0807 cells were treated with DiBAC_4_(3) after ZorO induction, the cells exhibited strong fluorescence, indicating that ZorO evokes membrane depolarization (Figure 1D). No significant fluorescence was detected from the cells without ZorO induction. When the *E. coli* cells were treated with propidium iodide (PI) after ZorO induction, PI-derived fluorescence was detected in most of the cells, revealing that the plasma membrane was damaged (Figure 1E). Therefore, ZorO localized in the inner membrane affects the membrane potential and integrity.

### 2.2. ZorO Affects Cell Viability Though Production of Hydroxyl Radicals

**Figure 2:**
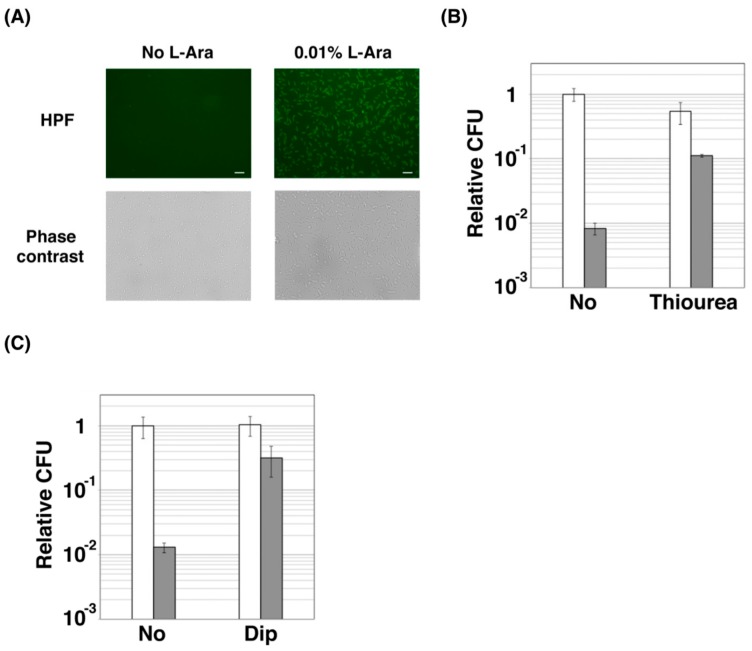
Effect of hROS on the ZorO toxicity: (**A**) TY0807 cells harboring pBAD24-FLAG-zorO were incubated with (right panels) or without (left panels) 0.01% L-Ara for 30 min and then treated with a reactive oxygen species indicator, hydroxyphenyl fluorescein (HPF), for 15 min. Upper or lower panels show HPF staining or phase-contrast images. Scale bar is 10 µm. (**B**,**C**) TY0807 cells harboring pBAD33-5′UTR-zorO were first treated with or without 250 mM Thiourea (B) or 2,2′-Bipyridl (Dip) (C) for 10 min and then treated with or without 0.05% L-Ara for 20 min. CFU in the absence of L-Ara and Thiourea or Dip is set to 1 and relative CFU was plotted. Gray or white bar graphs indicate the values of cells treated with or without L-Ara. An error bar shows a standard deviation deduced from at least three independent experiments.

Previous studies demonstrated that hydroxyurea and certain antibiotics cause cell death through the production of highly reactive oxygen species (hROS), such as hydroxyl radicals [26,27], although they are now controversial. Thus, the effect of ZorO on the level of hROS was also examined by using hydroxyphenyl fluorescein (HPF), which increases its fluorescence intensity upon binding to hROS. As shown in Figure 2A, ZorO-expressing *E. coli* TY0807 cells showed strong fluorescence, indicating that hROS is indeed produced in those cells. Whereas the proportion of viable cells were reduced to about 1% after ZorO induction (Figure 2B), treatment of the ZorO-expressing cells with thiourea (a hydroxyl radical scavenger) increased viable cells by 10-fold, suggesting that hydroxyl radicals are involved in the ZorO toxicity. Because hydroxyl radicals are produced from hydrogen peroxide and free ferrous iron though Fenton reaction, we then examined the effect of 2,2′-bipyridyl (Dip), a commonly used iron chelator for blocking Fenton reaction. As expected, a marked increase (~20-fold) in the proportion of viable cells was observed when the ZorO-expressing cells were treated with Dip (Figure 2C).

### 2.3. Amino Acid Residues of ALLRL Are Required for the Toxicity of ZorO

**Figure 3:**
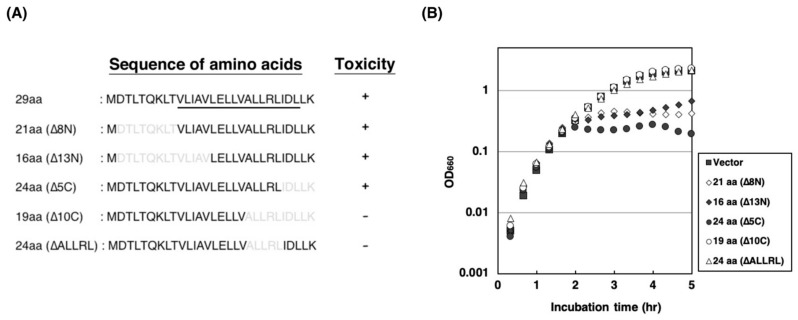
Toxicity of ZorO deletion mutants: (**A**) Amino acid sequences of wild-type and deletion mutants of ZorO are shown. Amino acids deleted from the ORF of ZorO are described in gray and the predicted transmembrane region is underlined. (**B**) TY0807 cells harboring pBAD24, pBAD24-zorO(∆8N), pBAD24-zorO(∆13N), pBAD24-zorO(∆5C), pBAD24-zorO(∆10C) or pBAD24-zorO(∆ALLRL) were treated with 0.2% L-Ara when the OD_660_ reached approximately 0.3. Symbols: ■: pBAD24, ◇: pBAD24-zorO(∆8N), ◆: pBAD24-zorO(∆13N), ●: pBAD24-zorO(∆5C), ○: pBAD24-zorO(∆10C), and △: pBAD24-zorO(∆ALLRL).

To identify the amino acids of ZorO required for the toxicity, we truncated the *N*- and the *C*-terminal residues (Figure 3A). As shown in Figure 3B, truncation of 8 and 13 *N*-terminal residues did not affect the toxicity of ZorO, indicating that the *N*-terminal 13 amino acids are dispensable. Likewise, the *C*-terminal 5 amino acids of ZorO appeared to be dispensable for its toxicity. On the other hand, when the *C*-terminal 10 amino acids were deleted, its toxicity was no longer observed (Figure 3B; open circles). The results indicate that Ala–Leu–Leu–Arg–Leu (ALLRL) spanning between positions 20 and 24 of ZorO are necessary for the toxicity. The ZorO-derived protein with the deletion of ALLRL did not inhibit the cell growth (Figure 3B; open triangles), confirming the importance of the five amino acids for the toxicity of ZorO.

### 2.4. The ALLRL Peptide Exhibits Antimicrobial Activity against Gram-Positive Bacteria and Candida

**Figure 4:**
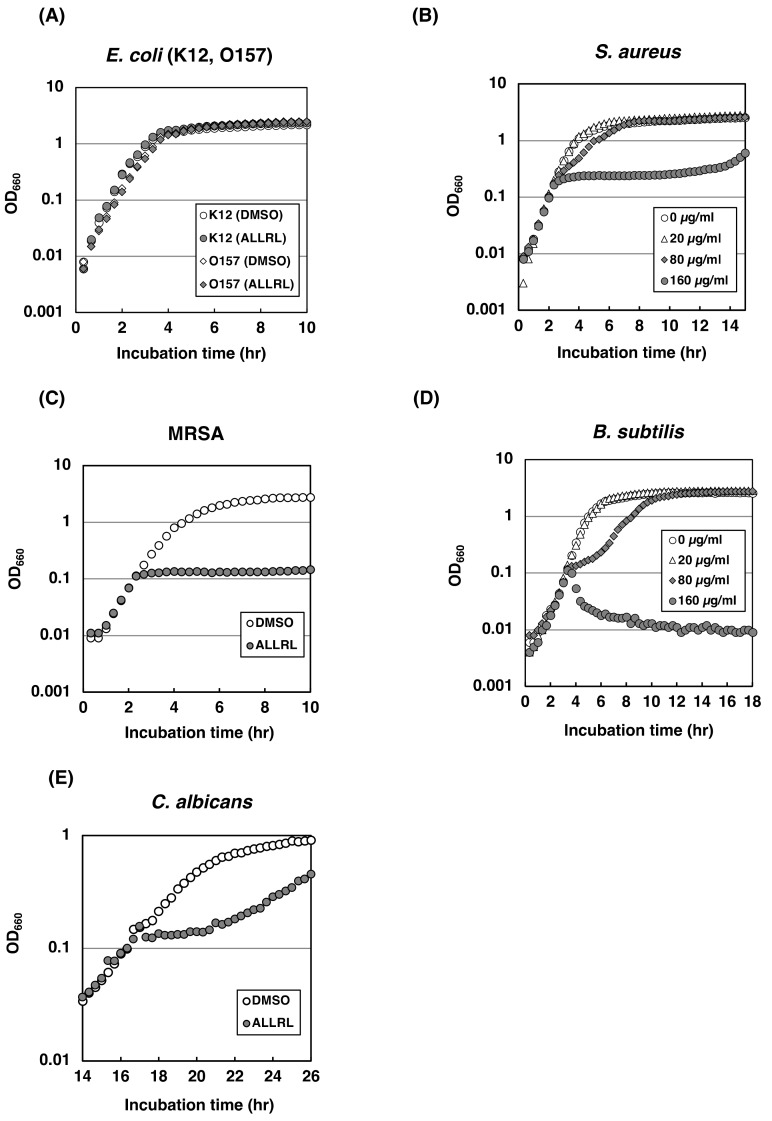

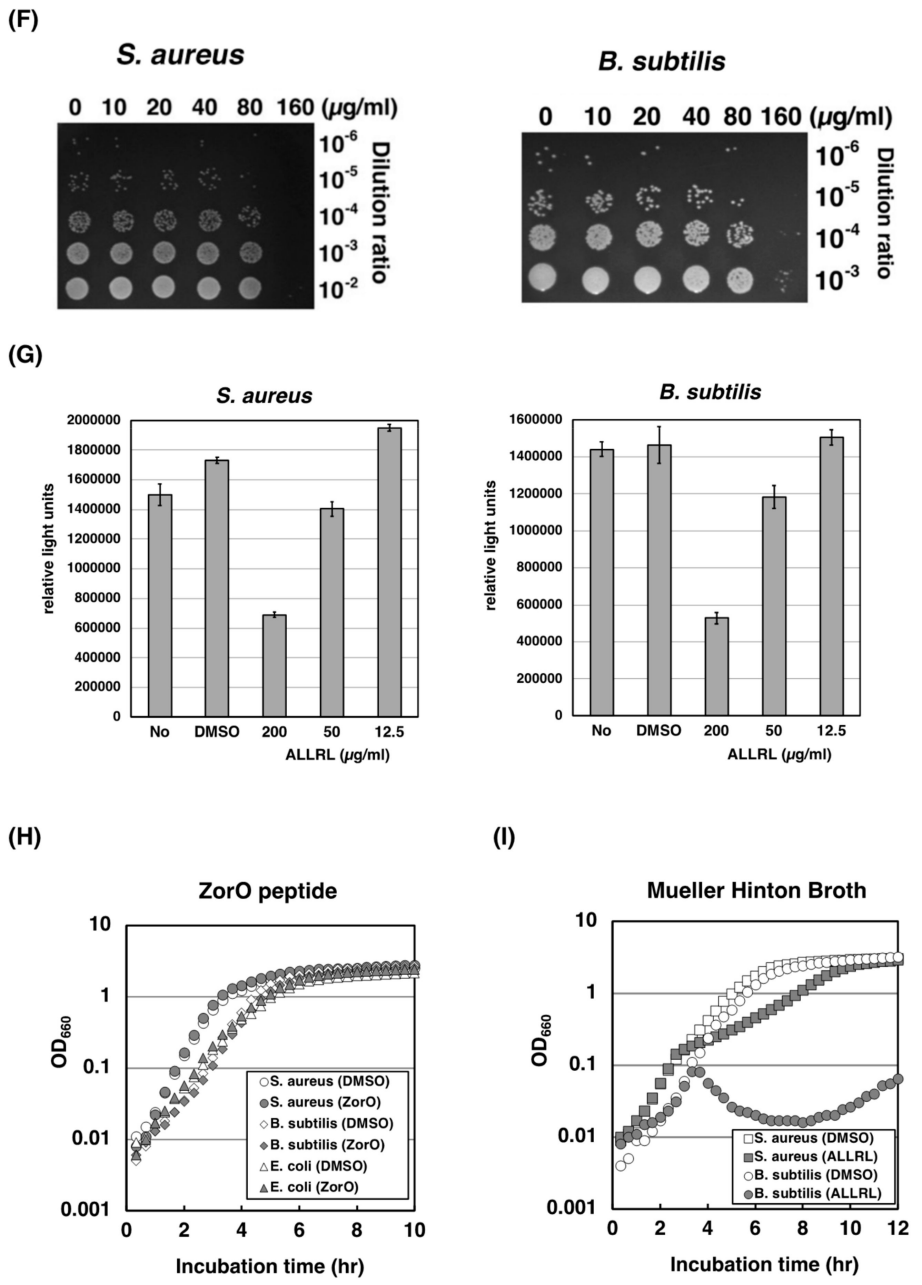
Antimicrobial activities of the ALLRL peptide (**A**) *E. coli* K12 BW25113 or O157:H7 were treated with 200 µg/mL of the ALLRL peptide or DMSO as a control when the OD_660_ reached approximately 0.2. (**B**) The methicillin-sensitive *S. aureus* was treated with 0, 20, 80, or 160 µg/mL of the ALLRL peptide when the OD_660_ reached approximately 0.2. (**C**) The methicillin-resistant *S. aureus* (MRSA) was treated with DMSO or 200 µg/mL of the ALLRL peptide when the OD_660_ reached approximately 0.2. (**D**) B*. subtilis* was treated with 0, 20, 80, or 160 µg/mL of the ALLRL peptide when the OD_660_ reached approximately 0.2. (**E**) *C. albicans* was treated with DMSO or 200 µg/mL of the ALLRL peptide when the OD_660_ reached approximately 0.1. (**F**) *S. aureus* (left panel) or *B. subtilis* (right panel) was grown in LB medium until the OD_660_ reached approximately 0.2, and then treated with the ALLRL peptide (0, 10, 20, 40, 80, or 160 µg/mL) for 1 h. Cell culture was serially diluted 10-fold; dilutes were spotted onto LB-agar plates and incubated at 37 °C overnight. (**G**) *S. aureus* (left graph) or *B. subtilis* (right graph) was treated with DMSO or the ALLRL peptide (12.5, 50, or 200 µg/mL) for 1 h or 30 min. Cell viability was determined using BacTiter-Glo™ Microbial Cell Viability Assay kit. The vertical axis indicates relative light units and the furthest left column is the values of cell culture without any treatment. (**H**) *S. aureus*, *B. subtilis*, or *E. coli* K12 BW25113 was treated with DMSO or 200 µg/mL of the full-length ZorO peptide when the OD_660_ reached approximately 0.1. (**I**) *S. aureus* or *B. subtilis* was grown in MHB medium and treated with DMSO or 100 µg/mL of the ALLRL peptide when the OD_660_ reached approximately 0.1.

The result that the stretch of five amino acid residues, ALLRL, was important for the ZorO toxicity prompted us to address the potential of the synthetic ALLRL pentapeptide as an antimicrobial. Here, the effects of the ALLRL peptide on the growth of various microorganisms were tested. As shown in Figure 4A, the ALLRL peptide by itself failed to exhibit toxic effects against *E. coli* K12 or O157:H7 even at the high concentration of 200 µg/mL. Interestingly, growth of Gram-positive bacteria, such as methicillin-sensitive and -resistant strains of *Staphylococcus aureus*, and *Bacillus subtilis*, was clearly inhibited immediately after the addition of the ALLRL peptide (160 µg/mL) (Figure 4B–D). Proportions of viable cells of the Gram-positive bacteria were decreased to about 10% and less than 0.01% by the addition of 80 and 160 µg/mL, respectively, of the ALLRL peptide (Figure 4F). Consistently, the ATP levels were also reduced by the ALLRL peptide in a dose-dependent manner (Figure 4G). Importantly, the effects of the ALLRL peptide were observed in both Meuller-Hinton Broth (No NaCl) and LB medium (171 mM NaCl) (Figure 4B,D,I), indicating that the peptide can exert the antimicrobial activity in the solution containing physiological salt concentrations. This is remarkable, given that some antibacterial peptides, such as human β-defensin-1, and a cathelin-associated peptide of human neutrophils, LL-37, significantly diminish their activities at physiological salt concentrations [6,7,8]. Surprisingly, the ALLRL peptide similarly inhibited the growth of a fungus, *Candida albicans* (Figure 4E). The synthetic peptide corresponding to the full-length ZorO showed no inhibitory effect on any microorganisms tested (Figure 4H).

### 2.5. The ALLRL Peptide Induces the Plasma Membrane Damage

**Figure 5:**
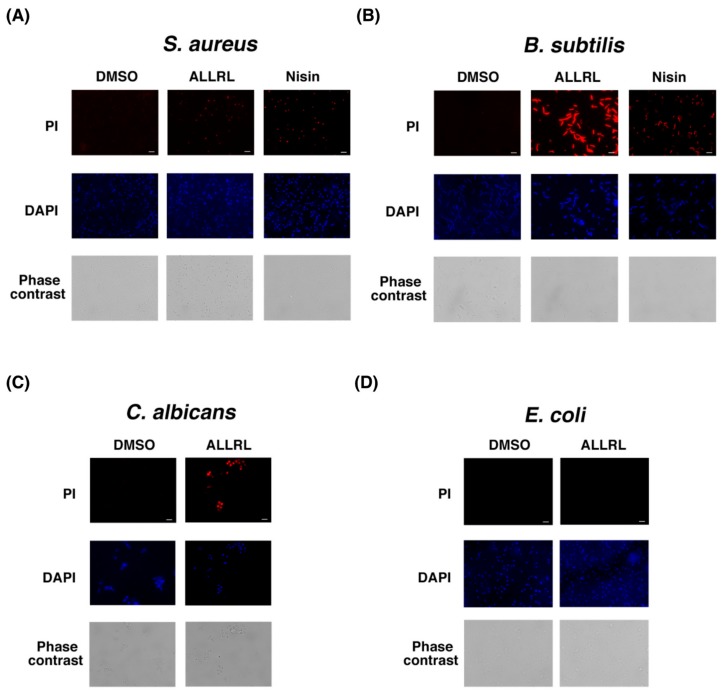
Cell membrane damage by the ALLRL peptide Overnight culture of *S. aureus* (**A**), *E. coli* TY0807 (**D**) or *B. subtilis* (**B**) was diluted 100-fold with LB medium and incubated at 37 °C for 2 h or 2 h 40 min. Ten microliters of DMSO or the ALLRL peptide (200 µg/mL) was added to 1 mL cells and incubated for 30 min. *S. aureus* or *B. subtilis* cell were also treated with nisin (N5764, Sigma-Aldrich) at the final concentration of 16 or 8 µg/mL for 20 min. *C. albicans* (**C**) was grown in 1 mL Sabouraud medium at 37 °C until the OD_660_ reached approximately 0.1 and then treated with 40 µL DMSO or the ALLRL peptide (200 µg/mL) for 40 min. Each cell was incubated with PI and DAPI. Upper, middle or lower panels show PI staining, DAPI staining, or phase contrast images. Scale bar is 10 µm.

In order to address the mechanisms by which the ALLRL peptide inhibits the growth of the Gram-positive bacteria and *Candida*, we examined the PI uptake by these microorganisms after treatment with the peptide. When *S. aureus*, *B. subtilis* and *C. albicans* cells were treated with the ALLRL peptide (200 µg/mL) for 30 min, the cells demonstrated a marked increase of PI uptake (Figure 5A–C). Nisin is an antimicrobial peptide with 34 amino acids produced by *Lactococcus lactis* subsp. *lactis* and induces the plasma membrane damage against Gram-positive bacteria [28]. *S. aureus* and *B. subtilis* cells treated with the nisin also exhibited the increase of PI uptake. These results suggest that, similar to the effects of the ZorO toxin expressed in *E. coli* cells, the ALLRL peptide damages the plasma membrane of Gram-positive bacteria and *Candida*, probably leading to the antimicrobial effects. Notably, the treatment of *E. coli* cells with the ALLRL peptide did not cause increased PI uptake (Figure 5D).

### 2.6. The ALLRL Peptide Has no Cytotoxicity Against Mammalian Cells

**Figure 6:**
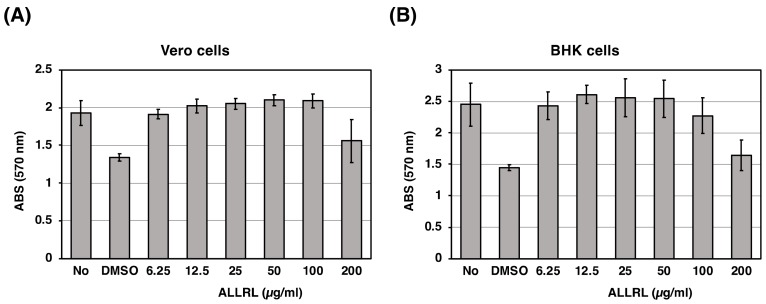
Cytotoxicity of the ALLRL peptide against mammalian cultured cells. Two mammalian cultured cells, Vero (**A**) or BHK (**B**), were treated with DMSO or the ALLRL peptide at final concentrations of 6.25, 12.5, 25, 50, 100, or 200 µg/mL for 24 h. Cell viability was measured by the MTT assay. The furthest left bar or the second bar from the left of each graph indicate the values of cells without any treatment or cells treated with 4% DMSO. The concentration of DMSO in growth medium containing 200 µg/mL of the ALLRL peptide is 4%. An error bar shows a standard deviation deduced from triplicate samples of each treatment group. Duplicate measurements were performed independently, similar results were obtained for each measurement, and a representative result was shown.

We also tested the effect of the ALLRL peptide on cultured mammalian cells, Vero and BHK. The ALLRL peptide was diluted two-fold serially with growth medium, both cells were incubated with the medium containing DMSO or the ALLRL peptide for 24 h and then cell viability was measured by the MTT assay. As shown in Figure 6, the ALLRL peptide failed to affect the viability of Vero and BHK cells, except that the peptide at the concentration of 200 µg/mL showed toxic effects. Both cells were shown to be sensitive to DMSO used as the solvent of the peptide (the second bar from the left of each graph). The concentration of DMSO in growth medium containing 200 µg/mL of the ALLRL peptide is 4%. Therefore, it is most likely that the apparent toxicity of the ALLRL peptide upon cells was caused by the effect of a relatively large amount of DMSO added. From these results, the ALLRL peptide is not toxic against mammalian cells.

## 3. Discussion

In this study, we first investigated the molecular mechanism of ZorO toxicity and next explored the possibility of using its derivatives as antimicrobial peptides. When ZorO expressed in *E. coli* cells, it localized to the inner membrane (Figure 1C) and induced cell membrane depolarization (Figure 1D) and damage (Figure 1E). Most type I TA toxins including ZorO comprise short (typically less than 60 amino acids) peptides rich in highly hydrophobic residues and harbor a putative transmembrane domain. Previous studies demonstrated that three *E. coli* TA toxins, TisB, GhoT and HokB, provoke cell membrane damage, which in turn lowers the ATP level [29,30,31]. Another study showed that, a *S. aureus* type I TA toxin, PepA1, also localizes to the cell membrane, and eventually triggers cell death [24]. Our data suggest that ZorO may act in a similar way for its toxicity. In addition, we demonstrated that ZorO promotes hROS production (Figure 2A) and that reducing the hydroxyl radical level with thiourea or 2,2′-bipyridyl partially restored the viability of cells treated with ZorO (Figure 2B,C). These results suggest that hydroxyl radicals play a key role in ZorO-mediated cell death. Cell membrane damage by ZorO may interfere with the electron transport chain, leading to the production of superoxide that is converted to hydrogen peroxide. At the same time, ZorO insertion may trigger iron uptake and increase the level of free ferrous iron. These changes may facilitate the Fenton reaction and consequently increase the production of hydroxyl radicals.

Nisin is an antimicrobial peptide with 34 amino acids produced by *Lactococcus lactis* subsp. *lactis* and has a broad spectrum of the activity against Gram-positive bacteria [28] and *C. albicans* [32]. In the United States, nisin is generally considered safe and has been approved for use in some processed-cheese spreads to prevent the proliferation of a pathogenic bacterium, *Clostridium botulinum* [33]. Its functional mechanism against Gram-positive bacteria has been well characterized. Nisin binds to the negatively-charged cell membrane through its positively charged amino acids and interacts with the cell wall precursor lipid II. Eight nisin peptides and four lipid II molecules assemble into a membrane pore with a diameter of 2.5 nm, which results in the leakage of cellular components and eventually cell death [28,34,35]. It is possible that the ALLRL peptide also interacts with Lipid II and forms a membrane pore.

Like nisin, the ALLRL peptide does not inhibit the growth of Gram-negative bacteria (Figure 4A). One possible explanation is that the outer membrane prevents these peptides from approaching the inner membrane. This idea may be supported by the result that electroporation is required for the antimicrobial activity of the type I TA toxin, Hok, against *E. coli* and *Pseudomonas putida* [36]. Indeed, a previous study demonstrated that the simultaneous treatment with nisin and a chelating agent, such as EDTA, inhibits the growth of Gram-negative bacteria [37]. This is probably because EDTA chelates magnesium ions and destabilizes the lipopolysaccharide layer of the outer membrane, which facilitates the entrance of nisin. We performed a similar experiment using the ALLRL peptide and EDTA but failed to observe an inhibitory effect on *E. coli* (unpublished data). Likewise, we could not see an inhibitory effect on *E. coli* O157 mutants, which lack lipopolysaccharides on the outer membrane (unpublished data). Therefore, the other mechanism would exist as to why the ALLRL peptide does not kill Gram-negative bacteria. It will give a hint for understanding its mechanism to test the bacterial spheroplast that lacks the outer membrane completely.

In conclusion, we discovered a small peptide to block the growth of Gram-positive bacteria including MRSA and of a fungus, *Candida* (Figure 4). To the best of our knowledge, the ALLRL peptide is the smallest antimicrobial peptide characterized so far. Also, most natural antimicrobial peptides reduce the activity in physiological salt concentrations but the ALLRL peptide exhibits the activity in the presence of 171 mM NaCl, which is suitable for antimicrobial drug development. Furthermore, the ALLRL peptide has no cytotoxicity against mammalian cultured cells, Vero and BHK (Figure 6). The ALLRL peptide, therefore, will be an attractive candidate for the development of an antimicrobial drug.

## 4. Materials and Methods 

### 4.1. Bacterial and Fungus Strains and Mammalian Cultured Cells

*Escherichia coli* K12 strains, TY0807 (*sup^0^ araD139 hsdR* ∆*lacX74 rpsL araD^+^*), BW25113 (*rrnB3* ∆ *lacZ4787 hsdR514* ∆ (*araBAD*)*567* ∆ (*rhaBAD*)*568 rph-1*) and *E. coli* O157:H7 strain (ATCC43888), were used [15,38,39]. Isolates of the methicillin-susceptible *Staphylococcus aureus*, the methicillin-resistant *S. aureus* (MRSA), *Bacillus subtilis* and *Candida albicans* used in this study were confirmed by sequencing of 16S or 18S rRNA. Primers used for sequencing are listed in Table 1. Luria-Bertani (LB) medium and Mueller Hinton Broth (MHB) were used for bacteria and Sabouraud medium (2% Glucose, 1% peptone, pH 5.5) for *C. albicans*. Vero cells from green monkey kidney were grown at 37 °C with 5% CO_2_ in Eagle’s Minimal Essential Medium (MEM) supplemented with 10% fetal bovine serum (FBS), 10 mM nonessential amino acid (NEAA; Life Technologies, Carlsbad, CA, USA) and Penicillin (1000 unit/mL)-streptomycin (100 µg/mL) (PS). BHK cells from the baby hamster kidney were grown at 37 °C with 5% CO_2_ in Dulbecco’s modified Eagle’s MEM supplemented with 10% FBS, 10 mM NEAA and PS.

### 4.2. Construction of Plasmids

Primers used for constructing plasmids are listed in Table 1. To construct pBAD24-zorO, pBAD24-FLAG-zorO, pBAD24-zorO(∆8N), pBAD24-zorO(∆13N), pBAD24-zorO(∆5C), or pBAD24-zorO(∆10C), a DNA fragment was amplified by PCR using *E. coli* O157:H7 DNA as a template and the primers, YO-371 and YO-57, YO-69 and YO-57, YO-370 and YO-57, YO-372 and YO-57, YO-371 and YO-79, or YO-371 and YO-373. The amplified fragments were digested with *Eco*RI and *Pst*I and ligated into the corresponding sites of pBAD24 [40]. pBAD24-zorO(∆ALLRL) were generated using a KOD-Plus-Mutagenesis kit (Toyobo) with pBAD24-zorO as the template and the primers YO-284 and YO-285. To generate pBAD33-5′UTR-zorO, a DNA fragment was amplified by PCR using O157:H7 DNA as a template and the primers YO-81 and YO-76, digested with *Eco*RI and *Hin*dIII, and ligated into the corresponding sites of pBAD18. Next, this plasmid (pBAD18-5′UTR-zorO) was digested with *Bam*HI and *Hin*dIII and ligated into pBAD33. All plasmid constructs were confirmed by sequencing with YO-82 primer.

### 4.3. Peptides

The peptides were synthesized commercially by Sigma-Aldrich and dissolved with DMSO at the final concentration of 10 or 20 mg/mL. The amino acid sequences of the peptides are MDTLTQKLTVLIAVLELLVALLRLIDLLK and ALLRL.

### 4.4. Growth of Bacteria and Fungus

TY0807 cells harboring pBAD24-FLAG-zorO, pBAD24-zorO(∆N8), pBAD24-zorO(∆N13), pBAD24-zorO(∆C5), pBAD24-zorO(∆C10), pBAD24-zorO(∆ALLRL) or empty pBAD24 vector were grown in LB medium supplemented with 50 µg/mL ampicillin (Amp). When the optical density at 660 nm (OD_660_) reached approximately 0.2–0.3, ZorO expression was induced with L-arabinose (L-Ara). Cell densities were monitored by the OD_660_ every 20 min using the biophotorecorder (Advantec TVS062CA). To detect the effects of the peptides, the methicillin-susceptible *S. aureus*, MRSA, *B. subtilis*, *E. coli* or *C. albicans* were grown in LB, MHB, or Sabouraud medium until the OD_660_ reached approximately 0.1–0.2; peptides were added at appropriate concentrations, and cell densities were monitored every 20 min. Duplicate or triplicate measurements were performed and similar results were obtained for each measurement. A representative result is shown for each figure.

### 4.5. Microbial Cell Viability Assay

TY0807 cells harboring pBAD24-FLAG-zorO were diluted 100-fold with LB medium supplemented with 50 µg/mL Amp, incubated for 2 h at 37 °C, and then treated with 0.02% L-Ara for 30 min. *S. aureus* or *B. subtilis* was grown in LB medium at 37 °C until the OD_660_ reached approximately 0.1 and then treated with the ALLRL peptide for 0.5 or 1 h. Cell viability was determined on triplicate samples of each treatment group using BacTiter-Glo™ Microbial Cell Viability Assay (Promega). Luminescence was measured using a luminescent reader (Lumat LB9501, Berthold).

### 4.6. Subcellular Fractionation and Western Blotting Analysis

TY0807 cells harboring pBAD24-FLAG-zorO were grown in 50 mL LB medium supplemented with 50 µg/mL Amp until the OD_660_ reached approximately 0.3 and then treated with 0.1% L-Ara for 10 min. Cells were harvested, washed once with the phosphate buffered saline (PBS), and suspended with 1 mL PBS. After cells were lysed by sonication, cell debris were removed by centrifugation at 5000× *g* for 5 min. The supernatant (0.8 mL) was centrifuged at 100,000× *g* for 45 min and the resulting supernatant was used as a cytoplasmic fraction and the pellet was used as a membrane fraction. To separate the inner and outer membranes, the pellet was resuspended with 0.4 mL PBS containing 0.4% Sarcosyl and incubated for 30 min at room temperature [23]. This mixture was recentrifuged at 100,000× *g* for 15 min and the supernatant was used as an inner membrane fraction. The pellet was resuspended with 0.4 mL PBS and used as an outer membrane fraction. Proteins from each fraction were separated though two 16% polyacrylamide gels containing tricine and SDS and detected either by Coomassie Brilliant Blue (CBB) or by Western blot using a mouse anti-FLAG M2 monoclonal antibody (Sigma-Aldrich) and a horseradish peroxidase-conjugated sheep anti-mouse IgG (GE Healthcare). FLAG-tagged ZorO were detected with Immobilon Western Chemiluminescent Substrate (Millipore) and a LAS-1000 image analyzer (Fujifilm). 

### 4.7. CFU Assay

Overnight culture of TY0807 cells harboring pBAD33-5′UTR-zorO were diluted 30-fold with LB medium supplemented with 30 µg/mL chloramphenicol and incubated at 37 °C for 110 min. Thiourea or 2,2′-Bipyridl (Dip) was added to the cells at the indicated final concentrations and incubated for 10 min. Cells were then treated with 0.05% L-Ara for 20 min, diluted with PBS, spread on LB-agar plates, and incubated at 37 °C overnight to count viable cells. *S. aureus* or *B. subtilis* were grown in LB medium until the OD_660_ reached approximately 0.1 and treated for 1 h with the ALLRL peptide at final concentrations of 0, 10, 20, 40, 80, or 160 µg/mL, respectively. Cell culture was serially diluted with PBS, and 5 µL of dilutes were spotted onto LB-agar plates and incubated at 37 °C overnight.

### 4.8. Microscopy

After treatment with L-Ara or the ALLRL peptide, each cell was recovered by the centrifugation at 3300× *g* for 5 min and suspended with 1 mL PBS. Twenty microliters of propidium iodide (P3566, Invitrogen) was added and incubated for 5 min at room temperature. After PI incubation, 0.5 µL of DAPI (4′, 6-diamidino-2-phenylindole dihydrochloride, 2.5 µg/mL) (D21490, Invitrogen) was added and further incubated for 5 min. After centrifugation, each cell pellet was resuspended with 100 µL PBS and cells were observed under the fluorescence microscopy (BZ-9000, KEYENCE). In experiments of the cell membrane depolarization and the production of highly reactive oxygen species, overnight cultures of TY0807 cells harboring pBAD24-FLAG-zorO were diluted 100-fold with LB medium containing Amp and incubated at 37 °C for 2 h. Cells were then treated with 0.01% L-Ara for 30 min, recovered by the centrifugation at 3300× *g* for 5 min and suspended with 1 mL PBS. Five microliters of DiBAC_4_(3) (B438, ThermoFisher Scientific) or 2 µL HPF (H36004, ThermoFisher Scientific) was added and incubated for 15 min at room temperature. After centrifugation, each cell pellet was resuspended with 100 µL PBS, and cells were observed with the fluorescence microscopy. Duplicate experiments were performed in figures, and similar results were obtained for each experiment. 

### 4.9. Cytotoxicity Assay (MTT Assay)

The cytotoxicity of the ALLRL peptide against Vero and BHK cells was determined by the Methylthiazolyldiphenyl-tetrazolium bromide (MTT) assay [41]. The ALLRL peptide was diluted two-fold serially with appropriate growth medium, and then cells at 70–90% confluency prepared in 96-well plates were treated with 4% DMSO or the ALLRL peptide at final concentrations of 6.25, 12.5, 25, 50, 100, or 200 µg/mL for 24 h at 37 °C in a humidified 5% CO_2_ incubator. After the medium was removed, 100 µL of 1 mg/mL MTT (M2128, Sigma-Aldrich) solution prepared in Eagle’s MEM containing 1% FBS was added to each well. After incubation for 4 h at 37 °C, the MTT solution was removed and 120 µL of isopropanol was added to dissolve the MTT crystals. After shaking the plates gently for 10 min, whereby crystals were completely dissolved, the absorbance at 560 nm was read on an iMark plate reader (BIO-RAD).

## Figures and Tables

**Table 1:** Oligonucleotides used in this study.

Primer Name	Sequence (5′–3′)	
*Construction of plasmids*		
YO-57	GCCTGCAGCTACTTCAACAAATCAATCAACCG	
YO-69	AGGAATTCACCATGGATTACAAGGATGACGACGATAAGATGGACTCGCTGACACAAAAG	
YO-76	GCAAGCTTCTACTTCAACAAATCAATCAACC	
YO-79	GCCTGCAGCTACAACCGTAACAGAGC	
YO-81	AGGAATTCGTTGGGACGTTGCGCCGGATCGA	
YO-82	AGATTAGCGGATCCTACCTG	
YO-284	CACTAATAACTCCAGTACGGCAATGAGCAC	
YO-285	ATTGATTTGTTGAAGTAGCTGCAGGCATGC	
YO-370	AGGAATTCACCATGGTGCTCATTGCCGTACTG	
YO-371	AGGAATTCACCATGGACACGCTGACAC	
YO-372	AGGAATTCACCATGCTGGAGTTATTAG	
YO-373	GCCTGCAGCTACACTAATAACTCCAG	
*Sequencing for bacterial 16S rRNA gene or C. albicans 18S rRNA gene*		
9F	GAGTTTGATCCTGGCTCAG	(for bacteria)
785F	GGATTAGATACCCTGGTAGTC	(for bacteria)
1510R	GGCTACCTTGTTACGA	(for bacteria)
YO-301	TATCTGGTTGATCCTGCCAGTAGTC	(for *C. albicans*)
YO-303	TTGATGCGTACTGGACCCAGCCG	(for *C. albicans*)
YO-304	GCGATAACGAACGAGACCTTAACC	(for *C. albicans*)
